# *Borrelia garinii* in Seabird Ticks (*Ixodes uriae*), Atlantic Coast, North America

**DOI:** 10.3201/eid1212.060448

**Published:** 2006-12

**Authors:** Robert P. Smith, Sabir Bin Muzaffar, Jennifer Lavers, Eleanor H. Lacombe, Bruce K. Cahill, Charles B. Lubelczyk, Allen Kinsler, Amy J. Mathers, Peter W. Rand

**Affiliations:** * Maine Medical Center Research Institute, South Portland, Maine, USA;; † Memorial University of Newfoundland, Saint John's, Newfoundland, Canada

**Keywords:** Borrelia garinii, Ixodes uriae, host-parasite interactions, seabirds, vectorborne disease, host-pathogen introduction, Atlantic Canada, research

## Abstract

*Borrelia garinii* is the most neurotropic of the genospecies of *B. burgdorferi* sensu lato that cause Lyme disease in Europe, where it is transmitted to avian and mammalian reservoir hosts and to humans by *Ixodes ricinus*. *B. garinii* is also maintained in an enzootic cycle in seabirds by *I. uriae*, a tick found at high latitudes in both the Northern and Southern Hemispheres. To determine whether *B. garinii* is present in seabird ticks on the Atlantic Coast of North America, we examined 261 *I. uriae* ticks by polyclonal antiborrelial fluorescent antibody. Ten of 61 ticks from Gull Island, Newfoundland, were positive for borreliae by this screen. Amplicons of DNA obtained by PCR that targeted the *B. garinii* rrs-rrla intergenic spacer were sequenced and matched to GenBank sequences for *B. garinii*. The potential for introduction of this agent into the North American Lyme disease enzootic is unknown.

## Abstract 2

TOC summary: The primary agent of neurotropic Lyme disease in Eurasia was found in seabird ticks in northeastern North America.

In Europe, Lyme disease is caused by 3 genospecies of Borrelia burgdorferi (i.e., B. afzelii, B. garinii, B. burgdorferi sensu stricto), while in North America only B. burgdorferi sensu stricto, the genospecies present in I. scapularis ticks, has been implicated in human disease. B. garinii, the most neurotropic of these 3 genospecies, causes most neurologic Lyme disease in Europe, including cases of meningopolyneuritis and, rarely, encephalomyelitis (*1**–**3*). The presence of multiple pathogenic genospecies that cause Lyme disease in Europe complicates serodiagnostic testing (*4*).

In Eurasia, B. garinii is transmitted to avian and rodent hosts and to humans by I. ricinus, the sheep or forest tick, and I. persulcatus, the taiga tick (*5**–**9*). I. uriae, the seabird tick, also maintains this agent in a "silent" enzootic cycle in seabirds at their nesting sites over a wide but discontinuous area (*10**–**13*). Although these 2 enzootic cycles are generally geographically and ecologically separate, interchange of B. garinii strains may occur at sites where both vectors coexist (*14*). The risk for seabird-associated strain types of B. garinii to cause Lyme disease, however, is not known (*15*).

Although B. garinii is present in seabird ticks in a nearly circumpolar distribution in both the Northern and Southern Hemispheres (*12**,**13*), including Alaska, the presence of B. garinii in I. uriae ticks at sites on the North Atlantic Coast has not previously been documented. We sought to determine whether B. garinii is present in ticks obtained from colonial seabird nesting sites on the Atlantic Coast of North America.

## Methods

**Figure 1:**
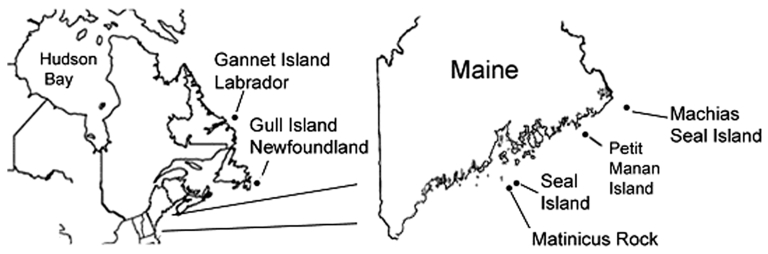
Locations in Maine (USA) and Atlantic Canada where *Ixodes uriae* ticks were collected.

Seabird researchers working at several sites on the Atlantic Coast in the United States and Canada (Figure 1) submitted I. uriae ticks to our laboratory for analysis (*16*). Ticks were identified to species, stage, and degree of engorgement (*17*). A subset of ticks was dissected, and midguts were screened for spirochetes by fluorescent microscopy by using a polyclonal antiborrelial antibody (*18*).

DNA was extracted from Borrelia-positive ticks by using the Qiagen DNeasy Tissue Kit (Qiagen, Valencia, CA, USA). DNA amplification was performed in a designated room with genus-specific primers that include the partial sequence of rrs-rrla intergenic spacer region as described by Bunikis et al. (*19*) with use of negative controls. Amplification products were viewed on a 1% agarose gel containing 0.5 μg/mL ethidium bromide. At a second laboratory, ticks positive by fluorescent antibody screen were prepared as above for DNA extraction, and PCR was performed by using primers directed at the 16S ribosomal DNA. Sequences of amplicons obtained at both laboratories were confirmed to be B. garinii by comparison with known sequences in the GenBank database.

## Results

I. uriae ticks submitted from 6 seabird colony sites and several hosts in the United States and Canada were processed (Table). Through 2005, 880 I. uriae ticks recovered primarily from Atlantic puffin chicks or their nests, were submitted from Maine sites, and another 383 were submitted from sites on the Atlantic Coast of Canada. Over 200 ticks from Maine sites and 61 ticks from Canadian sites off the coast of Newfoundland and Labrador were screened for borreliae by fluorescent microscopy.

**Table:** Submissions of *Ixodes uriae* from coastal Maine (USA) and Canada, 1996–2005

Site	Host species					On humans	Flag/drag sampling	Soil/litter sample
	Atlantic puffin (*Fratercula arctica*)	Murre (*Uria aalge*)/Razorbill (*Alca torda*)	Black guillemot (*Cepphus grille*)	Herring gull (*Larus argentatus*)	Common eider (*Somateria mollissima*)			
Machias Seal Island*	218					5	52	
Matinicus Rock*	258	23	25			210		
Petit Manan Island*	56		12					
Seal Island*	46							
Gannet Island†	111	88			8	72	31	
Gull Island‡	22			22		5	76	200

*Maine, USA. †Labrador, Canada. ‡Newfoundland, Canada.

**Figure 2:**
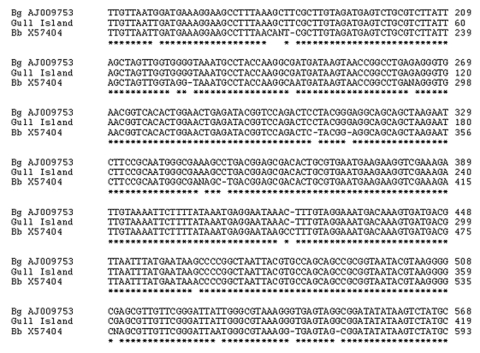
Alignments of 16S RNA sequences from GenBank: Bg AJ009753, *Borrelia garinii*; Bb X57404, *B. burgdorferi* strain B31, Gull Island, Newfoundland, Canada.

Spirochetes were detected only in ticks from Gull Island, Newfoundland (47° 15´N, 52° 46´W), where 9 of 22 adults and 1 of 39 nymphal ticks were positive. DNA was extracted from 2 of these ticks, and PCR showed a 1,900-base amplicon of the rrs-rrls intergenic spacer region that matched with B. garinii on comparison with GenBank sequences. Two additional ticks were examined in the laboratory of Sam Telford (Tufts University School of Veterinary Medicine, Grafton, MA, USA) by means of PCR targeting of 16S ribosomal DNA and again confirmed a match for B. garinii (GenBank bankit no. 800902 DG463373). Figure 2 shows the sequence from one of the ticks shown in an alignment with sequences from B. burgdorferi strain B31 and a representative B. garinii sample from GenBank.

## Discussion

The finding of B. garinii in I. uriae ticks from Gull Island, Newfoundland, adds to the known distribution of this agent and increases the likelihood that this agent is present in other colonial seabird nesting sites on the Atlantic Coast of North America, as is the case in Europe. The recent emergence of I. scapularis in coastal Maine and some Maritime Canadian sites (*20**,**21*) brings these 2 enzootic cycles of different genospecies of B. burgdorferi into proximate areas, although their ecologic settings differ. The public health importance of this finding depends on the probability of the introduction of B. garinii into emergent I. scapularis-vectored B. burgdorferi s.s. cycles and its potential maintenance in this cycle. The public health effects also depend on the pathogenic potential for human disease caused by seabird-associated strains of B. garinii (*15*).

The remote geographic and, at some sites, isolated topographic location of colonial seabird colonies provide significant barriers to the introduction of B. garinii into other vector ticks and their reservoir hosts. I. scapularis is dispersed to remote coastal islands of the North American Atlantic Coast during bird migration (*22**,**23*), but its establishment at these sites requires the presence of deer (*24**–**26*). With rare exception, deer are absent from sites with large seabird colonies, which are usually limited to offshore islands. Dispersal of infected I. uriae to proximate coastal areas by seabird hosts is unlikely because most species of seabirds parasitized by I. uriae are highly philopatric and forage at sea. One exception might be gulls, which may move between coastal and island sites. Passerine birds, which may forage near seabird colonies, provide another potential mechanism for dispersal of B. garinii, either through movement of infected I. uriae ticks or by serving as reservoir hosts of this agent. However, the frequency of parasitism of passerine birds by I. uriae ticks is unknown.

If B. garinii was introduced into I. scapularis ticks, its maintenance in this cycle would depend on the vector competence of I. scapularis for B. garinii, the reservoir competence of available hosts, and perhaps the population genetics and strain diversity of B. garinii (*14**,**27*). Although I. scapularis is vector-competent for transmission of B. garinii to rodents, its efficiency of transmission appears lower than for B. burgdorferi s.s (*28*). In addition, the vector competence of I. scapularis for seabird-associated strains of B. garinii has not been tested. The presence of similar ribotypes of B. garinii in I. ricinus ticks on a European island suggests that interchange of different B. garinii strains in different ecologic cycles may occur (*14*).

To determine the public health importance of B. garinii in seabird colonies along the North Atlantic coast, additional studies on the issues of dispersal, vector competence, and reservoir host competence are needed. All strain types of B. garinii may not be pathogenic for humans, and future studies should also address the potential for seabird-associated strains to cause disease.
